# Mitochondrial phylogenomics provides insights into the taxonomy and phylogeny of fleas

**DOI:** 10.1186/s13071-022-05334-3

**Published:** 2022-06-22

**Authors:** Yu Zhang, Yi-Tian Fu, Chaoqun Yao, Yuan-Ping Deng, Yu Nie, Guo-Hua Liu

**Affiliations:** 1 Research Center for Parasites & Vectors, College of Veterinary Medicine, Hunan Agricultural University, Changsha, 410128 Hunan province China; 2 Department of Biomedical Sciences and One Health Center for Zoonoses and Tropical Veterinary Medicine, Ross University School of Veterinary Medicine, P.O. Box 334, Basseterre, St. Kitts and Nevis

**Keywords:** *Pulex irritans*, *Ctenocephalides canis*, Mitochondrial genome, Phylogenetic analyses, Phylogenomics

## Abstract

**Background:**

Fleas (Insecta: Siphonaptera) are obligatory hematophagous ectoparasites of humans and animals and serve as vectors of many disease-causing agents. Despite past and current research efforts on fleas due to their medical and veterinary importance, correct identification and robust phylogenetic analysis of these ectoparasites have often proved challenging.

**Methods:**

We decoded the complete mitochondrial (mt) genome of the human flea *Pulex irritans* and nearly complete mt genome of the dog flea *Ctenocephalides canis*, and subsequently used this information to reconstruct the phylogeny of fleas among Endopterygota insects.

**Results:**

The complete mt genome of *P. irritans* was 20,337 bp, whereas the clearly sequenced coding region of the *C. canis* mt genome was 15,609 bp. Both mt genomes were found to contain 37 genes, including 13 protein-coding genes, 22 transfer RNA genes and two ribosomal RNA genes. The coding region of the *C. canis* mt genome was only 93.5% identical to that of the cat flea *C. felis*, unequivocally confirming that they are distinct species. Our phylogenomic analyses of the mt genomes showed a sister relationship between the order Siphonaptera and orders Diptera + Mecoptera + Megaloptera + Neuroptera and positively support the hypothesis that the fleas in the order Siphonaptera are monophyletic.

**Conclusions:**

Our results demonstrate that the mt genomes of *P. irritans* and *C. canis* are different. The phylogenetic tree shows that fleas are monophyletic and strongly support an order-level objective. These mt genomes provide novel molecular markers for studying the taxonomy and phylogeny of fleas in the future.

**Supplementary Information:**

The online version contains supplementary material available at 10.1186/s13071-022-05334-3.

## Background

Fleas (Insecta: Siphonaptera) are small, bilaterally flattened, wingless and diverse blood-feeding ectoparasites of mammals and birds [1]. They belong to the order Siphonaptera that includes more than 2500 valid species in 16 families [2, 3]. Fleas are one of the most common ectoparasites that serve as vectors of disease-causing agents, such as *Bartonella henselae* (cat scratch disease), *Francisella tularensis* (tularemia), *Rickettsia typhi* (murine typhus) and *Yersinia pestis* (plague) [4]. The human flea *Pulex irritans* and the dog flea *Ctenocephalides canis* have a worldwide distribution and are of high medical/veterinary importance [2, 5].

Accurate differentiation and identification of flea species are essential when diagnosing disease and in fundamental and applied research on these important ectoparasites [5–9]. *C. canis* and the cat flea *C. felis* have often been misidentified based on morphology because chaetotaxic variation is common [6]. In addition, the phylogeny of the order Siphonaptera within holometabolous insects is controversial. For example, while the monophyly of the order Siphonaptera is strongly supported by morphological features [2, 10], Tihelka et al. recently suggested that fleas should be treated as an infraorder of the order Mecoptera rather than as a separate order [11]. A very recent preprint has shown that fleas and mecopterans are sister groups, but the data were insufficient to distinguish whether the order Siphonaptera is sister to the order Mecoptera because the order Mecoptera is paraphyletic [12]. Thus, to date, the phylogenetic relationships of fleas remain unclear. The mitochondrial (mt) genome has been often used in systematics and phylogenetic studies across various taxonomic levels of different ectoparasites due to its nature of maternal inheritance, lack recombination, simple structure and rapid evolutionary rate [7–9, 13]. However, information on the mt genomes of fleas is limited [14–18], a deficiency which has greatly hindered the study of flea biology, genetics and phylogenetics. Therefore, there is a need to obtain more mt genomic data from more flea species. Such data would help to better understand the phylogenetic relationships of the order Siphonaptera, which notably include *P. irritans* (the primary vector of plague agents) and *C. canis* (vector of dipylidiasis pathogens).

The objectives of this study were: (i) to characterize the mt genomes of *P. irritans* and *C. canis*; (ii) to compare the mt genome sequences of *C. canis* with that of *C. felis* China isolate; and (iii) to assess the phylogenetic position of the order Siphonaptera within holometabolous insects.

## Methods

### Sample collection and DNA extraction

Adults of *P. irritans* and *C. canis* were collected from dogs brought by their owners to pet hospitals in Henan province, China. All animals were handled in strict accordance with good animal practice as defined by the relevant national and/or local animal welfare bodies, and all animal work was approved by the appropriate committee (No. 43321503). All fleas were stored in 70% ethanol immediately after collection and stored at − 80 °C until use. Prior to DNA extraction, the stored fleas were washed twice in physiological saline and air dried at room temperature. Genomic DNA was extracted from individual fleas using a Tissue DNA Kit (Promega, Madison, WI, USA) according to the manufacturer's instructions. DNA quantities was monitored on the Qubit 2.0 Fluorometer (Thermo Fisher Scientific, Waltham, MA, USA). Species identification of individual fleas was molecularly determined by PCR-based sequencing of the nuclear elongation factor 1 α (EF-1α) and mt* cox*2 genes as previously described [7, 13]. The sequences of EF-1α and the *cox*2 genes of human fleas had 100% and 98% identity to those of *P. irritans* originated from the USA (GenBank accession nos. AF423871 and MF136072), respectively. The sequences of EF-1α and the *cox*2 genes of dog fleas had 99% and 100% similarity to those of dog fleas from the Czech Republic and Hungary (GenBank accession nos. MG586747 and MG637389), respectively. These data collectively confirm that these fleas are *P. irritans* and *C. canis*, respectively.

### Sequencing, assembling and verification

For *P. irritans*, a genomic DNA library of approximately 350 bp was constructed and used for high-throughput sequencing on the NovaSeq 6000 platform (Agilent Technologies, Santa Clara, CA, USA) with 250-bp paired-end reads. The raw reads in the FASTQ format were exported and then cleaned by removing adaptor reads, highly repetitive reads and ‘N’-rich reads using the fastp program [19]. The resulting clean reads were de novo assembled using the Velvet algorithm in Geneious Prime 2021.2.2 [20] based on the obtained *cox*2 sequence. The criteria were 1% mismatch, a maximum gap of 5 bp and a minimum overlap of 150 bp. A complete mt genome of *P. irritans* was assembled and was further confirmed by PCR using three pairs of specific primers (Additional file 5: Table S1) for all gene-coding regions.

For *C. canis*, specific primers (Additional file 5: Table S2) were designed based on cat flea *C. felis* China isolate (Genbank accession number: MW420044) [18]. The seven overlapping PCR amplicons covered regions between the AT region and *nad*2(approx. 1.4 kb), between transfer RNA (tRNA)-Ile and *cox*1 (approx. 1.7 kb), between *cox*1 and *cox*2 (approx. 1.9 kb), between *cox*2 and *cox*3 (approx. 2.0 kb), between *cox*3 and *nad*5 (approx. 2.5 kb), between *nad*5 and *cyt*b (approx. 4.0 kb) and between* cyt*b and the AT region (approx. 3.9 kb). The PCR mix (reaction volume: 25 μl) included 10.5 μl ddH_2_O, 0.5 μl each of the sense and antisense (2 μM) primer, 12.5 μl Master mix (Takara Bio, Kusatsu, Shiga, Japan) and 1 μl genomic DNA. The thermal cycling program consisted of an initial denaturing at 94 °C for 1 min, followed by 35 cycles of 98 °C for 10 s, 45–65 °C for 40 s depending upon the primers used, 68 °C for 4 min, with a final elongation for 8 min at 72 °C. Purified PCR amplicons were sequenced in both directions (Beijing Genomics Institute, Shenzhen, China).

### Genome annotation

The assembled mt genomes were annotated using MITOS webservers [21]. The boundaries of the protein-coding genes and ribosomal RNA (rRNA) genes were discerned by alignment with the homologs of *C. felis* China isolate using MAFFT 7.122 [22]. tRNA genes were annotated using ARWEN [23] and tRNAscan-SE [24]. Nucleotide composition, amino acid sequences of individual protein-coding genes and codon usage were analyzed using MEGA X [25].

### Phylogenetic analysis

The representative mt genome sequences of holometabolous insects, along with *Philaenus spumarius* (GenBank accession number: NC005944) as an outgroup [26], were obtained from GenBank for phylogenetic analysis (Additional file 5: Table S3). Individual amino acid sequences of all 13 mt protein-coding genes were aligned using MAFFT 7.122. The aligned sequences were then concatenated to form a single dataset. Ambiguous positions were excluded using Gblocks 0.91b [27] with the option for a less stringent selection.

Phylogenetic trees were reconstructed using Bayesian inference (BI) in MrBayes 3.2.6 [28] and by maximum likelihood (ML) in IQ-TREE v.2.1.3 [29]. For BI analysis, the alignment was partitioned by gene, and the MtArt model of amino acid evolution was selected as the most suitable model of evolution by the ProtTest 3.4 [30] based on the Akaike information criterion (AIC). As the MtArt model is not implemented in the current version of MrBayes, an alternative model, MtREV, was used in the Bayesian analysis. Four independent Markov chains were run for 10 million generations. The trees were sampled every 1000 generations with the first 25% discarded as burn-in. For the ML analysis, the optimal partitioning scheme and the best evolutionary model for each partition was selected under the corrected AIC in IQ-TREE. The ML tree was selected with IQ-TREE by an ultrafast bootstrap approximation approach with 10,000 replicates. The phylogenetic trees were visualized using FigTree v.1.42.

## Results

### General features of the mt genomes

**Fig. 1:**
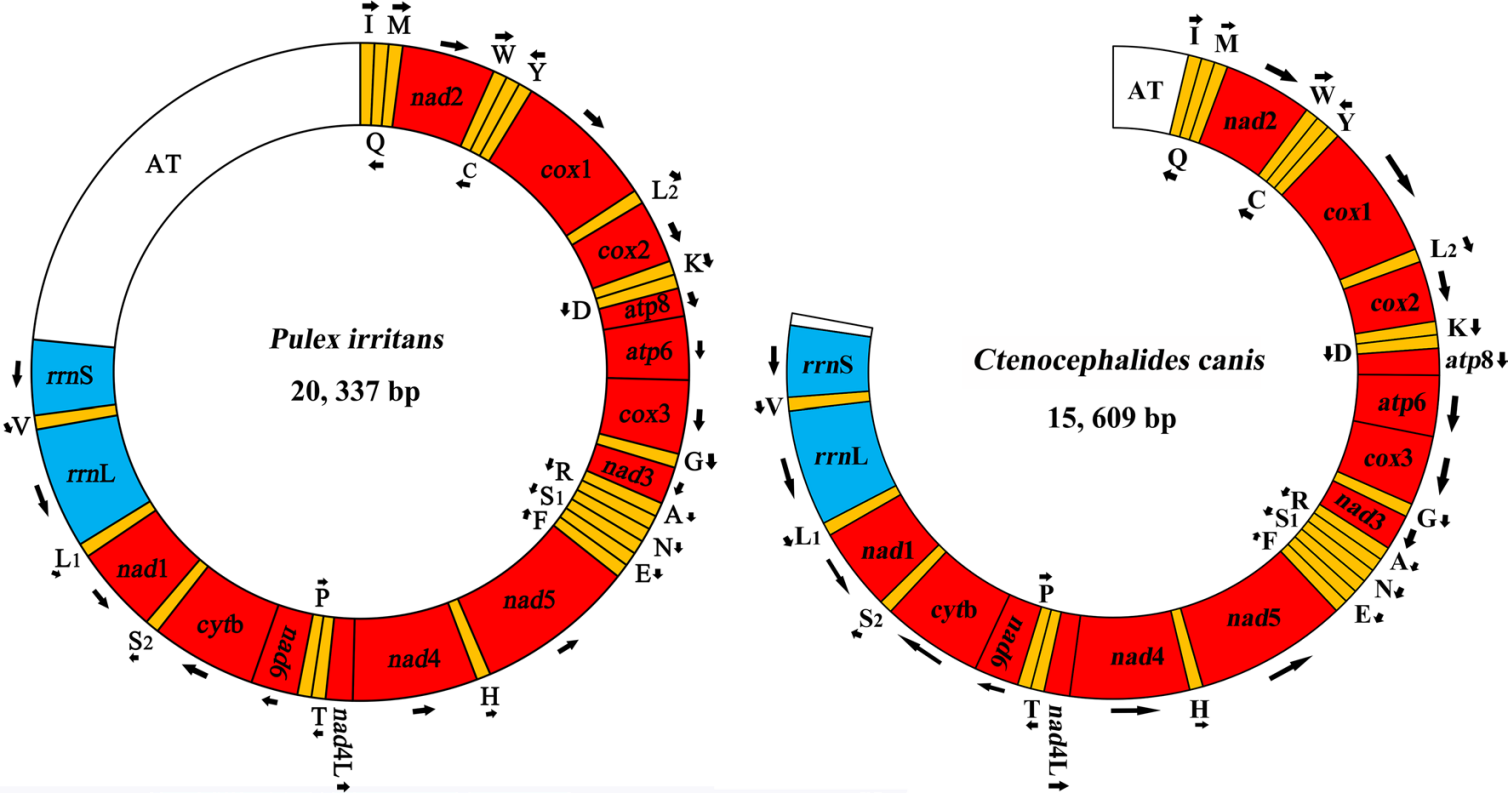
The complete mt genome of human flea *Pulex irritans*, and the nearly complete mt genome (except for partial non-coding region) of dog flea *Ctenocephalides canis*. The names and transcription orientation of the genes are indicated in the coding region. Protein-coding and rRNA genes are indicated using standard nomenclature. tRNA genes are indicated with the one-letter code of their corresponding amino acids. There are two tRNA genes for leucine: L_1_ for codons CUN and L_2_ for UUR; and two tRNA genes for serine: S_1_ for codons AGN and S_2_ for UCN

A total of 6 Gb of Illumina short-read sequence datasets was generated for the mt genome of *P. irritans*, resulting in 13,123,958 × 2 clean reads. The complete mt genome with 20,337 bp in size was submitted to GenBank with accession no. ON100828 (Fig. 1). It was further verified by three PCR amplicons covering the entire gene-coding region (Additional file 1: Figure S1). The nearly complete mt genome, with the exception of the partial non-coding region of *C. canis* (GenBank accession no. ON109770), was 15,609 bp (Fig. 1). Again, this structure was confirmed by seven overlapped PCR amplicons (Additional file 2: Figure S2). Both mt genomes contained 37 genes, including 13 protein-coding genes (*cox*1-3, *nad*1-6, *nad*4L, *atp*6, *atp*8 and *cyt*b), two rRNA genes and 22 tRNA genes (Table 1; Fig. 1). Twenty-three genes were on the heavy strand, and the rest were on the light strand (Table 1). The genes in the mt genome of *P. irritans* overlapped in 10 locations, comprising 37 bp in total, with overlaps of 1–13 bp per location. There were 10 intergenic regions consisting of a total of 188 bp, with the longest intergenic region located between tRNA-Met and *nad*2 (Table 1). Similarly, the mt genome of *C. canis* overlapped at eight locations, comprising 36 bp in total, with overlaps of 1–13 bp per location, and had nine intergenic regions ranging from 1 to 38 bp (Table 1). The nucleotide composition of *P. irritans* was: A = 5658 bp (38.4%), T = 5974 bp (40.6%), G = 1207 bp (8.2%) and C = 1892 bp (12.8%); this was similar to the nucleotide composition of *C. canis*: A = 5783 bp (39.5%), T = 5922 bp (40.5%), G = 1173 bp (8.0%) and C = 1759 bp (12.0%).

**Table 1:** Organization of the mitochondrial genomes of human flea *Pulex irritans* and dog flea *Ctenocephalides canis*

Gene/region	Positions		Strand		Size (bp)		Number of aa^a^		Ini/Ter codons^b^		Anticodon		Intergenic nucleotides	
	*Pi*	*Cc*	*Pi*	*Cc*	*Pi*	*Cc*	*Pi*	*Cc*	*Pi*	*Cc*	*Pi*	*Cc*	*Pi*	*Cc*
tRNA-Ile (I)	3235–3297	841–904	H	H	63	64					GAT	GAT	0	
tRNA-Gln (Q)	3406–3338	970–902	L	L	69	69					TTG	TTG	40	− 3
tRNA-Met (M)	3472–3539	1009–1076	H	H	68	68					CAT	CAT	65	38
*nad*2	3540–4539	1081–2078	H	H	1000	998	333	332	ATT/T	ATG/TA			0	4
tRNA-Trp (W)	4540–4604	2019–2143	H	H	65	65					TCA	TCA	0	0
tRNA-Cys (C)	4657–4598	2196–2136	L	L	60	61					GCA	GCA	− 7	− 8
tRNA-Tyr (Y)	4721–4658	2261–2198	L	L	64	64					GTA	GTA	0	1
*cox*1	4755–6256	2274–3796	H	H	1502	1523	500	507	ATT/TA	TTT/TA			33	12
tRNA-Leu^UUR^ (L_2_)	6257–6321	3797–3860	H	H	65	64					TAA	TAA	0	0
*cox*2	6322–7002	3861–4539	H	H	681	679	226	226	ATG/TAA	ATG/T			0	0
tRNA-Lys (K)	7005–7075	4540–4610	H	H	71	71					CTT	CTT	2	0
tRNA-Asp (D)	7075–7139	4610–4673	H	H	65	64					GTC	GTC	− 1	− 1
*atp*8	7140–7301	4674–4838	H	H	162	165	53	54	ATT/TAA	TTG/TAA			0	0
*atp*6	7289–7969	4826–5503	H	H	681	678	226	225	TTG/TAA	TTG/TAA			− 13	− 13
*cox*3	7969–8751	5503–6285	H	H	783	783	260	260	ATG/TAA	ATG/TAA			− 1	− 1
tRNA-Gly (G)	8752–8814	6286–6347	H	H	63	62					TCC	TCC	0	0
*nad*3	8815–9163	6348–6696	H	H	349	349	116	116	ATC/T	ATT/T			0	0
tRNA-Ala (A)	9164–9227	6697–6760	H	H	64	64					TGC	TGC	0	0
tRNA-Arg (R)	9228–9289	6761–6819	H	H	62	59					TCG	TCG	0	0
tRNA-Asn (N)	9291–9355	6821–6885	H	H	65	65					GTT	GTT	1	1
tRNA-Ser^AGN^ (S_1_)	9355–9423	6884–6943	H	H	69	60					TCT	TCT	− 1	− 2
tRNA-Glu (E)	9422–9489	6944–7006	H	H	68	63					TTC	TTC	− 2	0
tRNA-Phe (F)	9552–9487	7070–7007	L	L	66	64					GAA	GAA	− 3	0
*nad*5	11,269–9562	8781–7071	L	L	1708	1711	569	570	ATG/T	ATT/T			9	0
tRNA-His (H)	11,333–11,271	8843–8782	L	L	63	62					GTG	GTG	1	0
*nad*4	12,660–11,334	10,179–8844	L	L	1327	1336	442	445	ATG/T	ATG/T			0	0
*nad*4L	12,944–12,654	10,460–10,173	L	L	288	288	95	95	ATG/TAG	ATG/TAA			− 7	− 7
tRNA-Thr (T)	12,947–13,010	10,463–10,525	H	H	64	63					TGT	TGT	2	2
tRNA-Pro (P)	13,078–13,010	10,590–10,526	L	L	69	65					TGG	TGG	− 1	0
*nad*6	13,080–13,586	10,593–11,093	H	H	507	501	168	166	ATT/TAA	ATT/TAA			1	2
*cyt*b	13,686–14,717	11,093–12,224	H	H	1132	1132	377	377	ATG/T	ATG/T			− 1	− 1
tRNA-Ser^UCN^ (S_2_)	14,718–14,783	12,225–12,290	H	H	66	66					TGA	TGA	0	0
*nad*1	15,747–14,818	13,248–12,319	L	L	930	930	309	309	ATT/TAG	ATG/TAA			34	28
tRNA-Leu^CUN^ (L_1_)	15,810–15,748	13,312–13,251	L	L	63	62					TAG	TAG	0	2
*rrn*L	17,104–15,811	14,612–13,313	L	L	1294	1300							0	0
tRNA-Val (V)	17,171–17,105	14,679–14,613	L	L	67	67					TAC	TAC	0	0
*rrn*S	17,964–17,172	15,477–14,680	L	L	793	798							0	0
AT-loop reg*ion*	1–3234;17,965–20,337	1–840;15,478–15,609			5607	972								

*aa* Amino acid, *Cc* *Ctenocephalides canis*, *Pi* *Pulex irritans*

^a^The inferred length of the aa sequence of 13 protein-coding genes

^b^Ini/Ter codons: initiation and termination codons

### Annotation

All protein-coding genes in the *P. irritans* mt genome used ATT, ATG, TTG or ATC as a start codon, and TAA, TAG, TA or T as a stop codon (Table 1). In the *C. canis* mt genome, ATT, ATG, TTG or TTT were used as start codons, and ATA, T or TA were used as stop codons (Table 1). The large subunit of rRNA gene (*rrn*L) was located between tRNA-Leu^CUN^ (L_1_) and tRNA-Val(V), and the small subunit of rRNA gene (*rrn*S) was located between tRNA-Val (V) and non-coding region (Table 1; Fig. 1). The *rrn*L and *rrn*S genes of *P. irritans* were 1294 and 793 bp, respectively, and those of *C. canis* were 1300 and 798 bp, respectively (Table 1). A + T contents of *rrn*L and *rrn*S of *P. irritans* were 82.8% and 82.1%, respectively, and those of *C. canis* were 83.5% and 81.8%, respectively. The 22 tRNA genes of both *P. irritans* and *C. canis* ranged in length from 60 to 71 bp (Table 1). The predicated secondary structures of 22 tRNA genes (Additional file 3: Figure S3; Additional file 4: Figure S4) were similar to those of *C. felis*, as previously reported [18].

### Comparative analyses of the mt genomes of *C. canis* and *C. felis* China isolate

The coding regions of the mt genome of *C. canis* were in total 1 bp shorter than those of the *C. felis* China isolate (14,638 bp). The coding regions of both mt genomes were arranged in the same way. There were 6.5% nucleotide sequence differences among all genes between *C. canis* and the *C. felis* China isolate. The *nad*6 gene showed the greatest variation in nucleotide composition (9.9%), whereas the *rrn*S gene showed the least (3.0%) (Table 2). We also compared the predicted amino acid sequences of individual mt genes of *C. canis* with those of the *C. felis* China isolate (Table 2). The differences ranged from 0.4% to 10.2%, with COX2 being the most conserved protein and NAD6 the least conserved (Table 2). The sequence variation of the 22 tRNA genes was 3.2% between *C. canis* and the *C. felis* China isolate. The *rrn*L and *rrn*S genes showed 4.2% and 3.0% sequence differences, respectively. Taken together, the mt genome datasets presented here confirm that *C. canis* and *C. felis* represent distinct flea species.

**Table 2:** Nucleotide and/or predicted amino acid sequence differences in mitochondrial genes between *C. canis* and *C. felis* upon pairwise comparison

Gene/region	Nt sequence length		Nt difference (%)	Number of aa		aa difference (%)
	*C. canis*	*C. felis*	*C. canis/C. felis*	*C. canis*	*C. felis*	*C. canis/C. felis*
*nad*2	998	1004	6.5	332	334	5.1
*cox*1	1523	1523	7.4	507	507	0.6
*cox*2	679	679	4.7	226	226	0.4
*atp*8	165	162	7.9	54	53	5.6
*atp*6	678	678	9.1	225	225	4.0
*cox*3	783	783	9.8	260	260	5.8
*nad*3	349	343	8.9	116	114	7.8
*nad*5	1711	1706	7.4	570	568	5.8
*nad*4	1336	1336	5.5	445	445	2.9
*nad*4L	288	288	4.2	95	94	1.1
*nad*6	501	504	9.9	166	167	10.2
*cyt*b	1132	1132	8.4	377	377	4.0
*nad*1	930	930	7.5	309	309	3.2
All 22 tRNA	1412	1415	3.2	–	–	–
*rrn*L	1300	1301	4.2	–	–	–
*rrn*S	798	785	3.0	–	–	–

*nt* Nucleotide

### Phylogenetic relationships

**Fig. 2:**
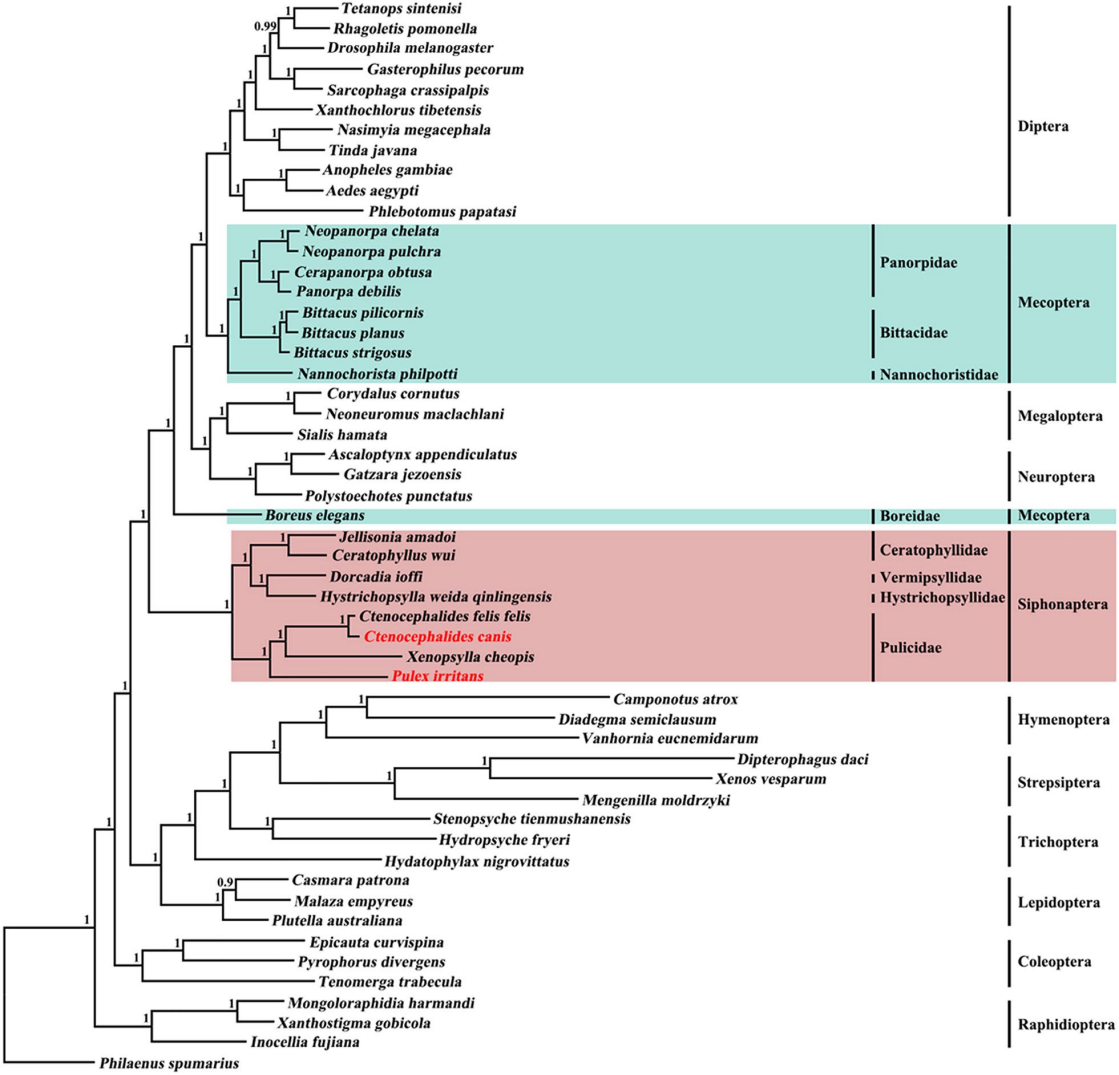
Phylogenetic relationships among 52 species of Endopterygota insects inferred from Bayesian inference (BI) analysis of deduced amino acid sequences of 13 mt proteins. *Philaenus spumarius* (GenBank accession number: NC005944) was used as the outgroup. Bayesian posterior probability (Bpp) values are indicated at nodes. Details of mt genomes, including accession numbers, are included in Additional file 5: Table S3

**Fig. 3:**
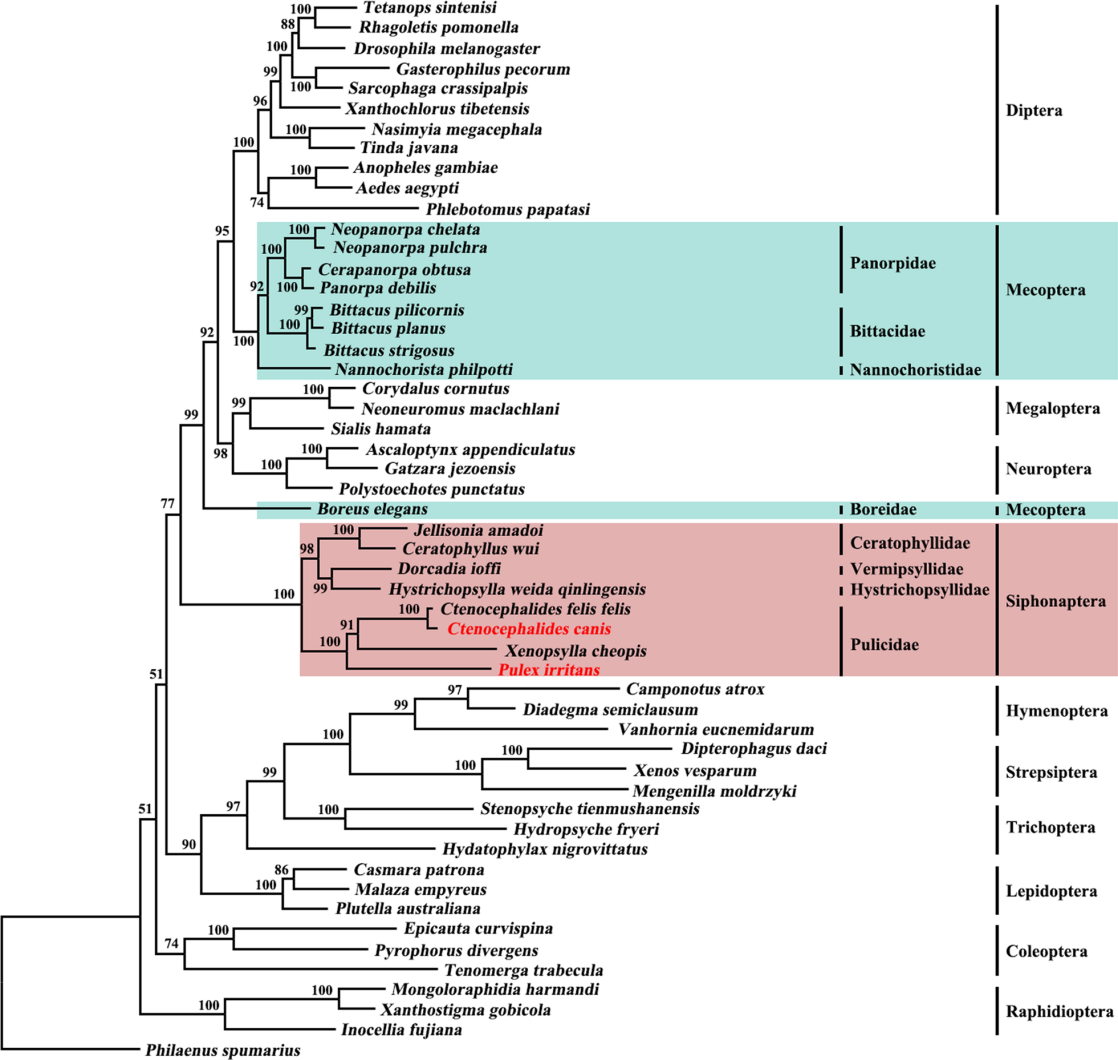
Phylogenetic relationships among 52 species of Endopterygota insects inferred from maximum likelihood (ML) analysis of deduced amino acid sequences of 13 mt proteins. *Philaenus spumarius* (GenBank accession number: NC005944) was used as the outgroup. Ultrafast bootstrap approximation (UFBoot) values are indicated at nodes. Details of mt genomes, including accession numbers, are included in Additional file 5: Table S3

Two phylogenetic analyses of the concatenated amino acid sequences of all 13 proteins encoded by the mt genome showed that eight flea species used to construct the phylogenetic trees in this study grouped together (Figs. 2, 3). Our phylogenomic analysis further showed that the order Siphonaptera was monophyletic, as strongly supported by the calculated Bayesian posterior probability (Bpp) value (Bpp = 1.0) in the BI analysis and UFBoot value (UFBoot = 1.0) in the ML analysis. The *C. canis* was more closely related to *C. felis* than to the other members of the family Pulicidae (Figs. 2, 3). In addition, Siphonaptera is a sister group of orders Diptera + Mecoptera + Megaloptera + Neuroptera, with a strong support in the BI analysis (Bpp = 1.0) and a moderate support in the ML analysis (UFBoot = 77) (Figs. 2, 3). In contrast, the order Mecoptera was not monophyletic (Figs. 2, 3).

## Discussion

Fleas are the most common ectoparasites infesting dogs and cats worldwide, and they can also severely affect human health. The accurate identification and differentiation of flea species has important implications for the diagnosis of flea-borne diseases and the prevention and control of fleas and these diseases. Flea species such as *C. canis* and *C. felis* are usually identified by morphology [31]. However, the identification and differentiation of closely related flea species are often technically challenging [6].

In the present study, characterization of the mt genomes of both *P. irritans* and *C. canis* provides a complementary tool to investigate the genetic composition of flea species. Previous studies have used genetic markers in the internal transcribed spacer 1 and 2 (ITS-1 and ITS-2, respectively) regions of nuclear rDNA [32] and mt *cox*1 and *cox*2 genes [13] in the molecular identification of *P. irritans* and *C. canis*. In addition, molecular and phylogenetic analyses have detected two cryptic *P. irritans* species [33]. However, mt genes *cox*1 and *cox*2 are better suited for such studies than the ITS-1 and ITS-2 regions owing to their high level of nucleotide diversity [5].

In the present study, characterization of the mt genome of *C. canis* provides a molecular marker for enriching comparative analyses in flea taxa. Comparison between the mt genomes of *C. canis* and *C. felis* revealed a sequence variation of 6.5% across the coding region of these genomes. This level of nucleotide sequence difference (6.5%) is high. Previous studies of other insects have detected a similar difference in their mt genomes. For example, the difference in the nucleotide sequences of the coding region between *Neochauliodes sinensis* (GenBank accession number: MW642295) and *N. meridionalis* was 6.1% (GenBank accession number: MW642293), and the difference between *N. rotundatus* (GenBank accession number: MW642294) and *N. sparsus* was 6.2% (GenBank accession number: MW642296) [34]. In the present study, a clean genetic distinctiveness was detected between *C. canis* and *C. felis* China isolate, but host affiliation is not strict [4, 6]. Cross-infection of *C. canis* has often been found in cats, and in many geographical regions *C. felis* has been more often found on dogs than *C. canis* on dogs [4, 6]. Despite the compelling evidence of genetic distinctiveness between *C. canis* and *C. felis* China isolate, further study is required to confirm the genetic and phylogenetic relationships among species or subspecies of *Ctenocephalides* using larger numbers of specimens from broader geographical locations. Simultaneously, detailed morphological redescriptions of these fleas are needed.

Our characterization of the mt genomes of *P. irritans* and *C. canis* in the present study also stimulates reassessing the phylogenetic position of the order Siphonaptera among the holometabolous insects using mt genomic datasets. Phylogenetic analyses using a small number of genes, including* 18S* and* 28S* rRNA, *cox*2 and EF-1α have demonstrated that the order Mecoptera is paraphyletic. The order Siphonaptera nests within the order Mecoptera as a sister group to the family Boreidae, and the obscure family Nannochoristidae is placed as a sister group to Boreidae + Siphonaptera [10, 35–37]. Recently, the results of an analysis similar to the one presented here using the largest molecular dataset to date indicated fleas as a nested group within the order Scorpionflies as a sister group to the enigmatic Southern Hemisphere family Nannochoristidae [11]. However, phylogenomic analyses of both nucleotide and amino acid sequences of 1478 protein-coding genes robustly and congruently lead to the conclusion that both Siphonaptera and Mecoptera are monophyletic [38]. Nevertheless, the results of a phylogenetic analysis using large-scale transcriptomic data provide strong support that fleas and mecopterans together are the sister groups of flies, although based on these results it is not possible to resolve whether Siphonaptera is a sister group to the monophyletic Mecoptera [12]. These controversial results show that the phylogeny of fleas among insects has proved challenging to resolve.

The results of the phylogenomic analysis performed in the present study support the hypothesis that the order Siphonaptera is monophyletic (Figs. 2, 3). They also revealed a sister relationship between Siphonaptera and orders of Diptera + Mecoptera + Megaloptera + Neuroptera. However, in the current study we did not establish the monophyly of Mecoptera, which is consistent with current decades-long controversy on the monophyly of Mecoptera involving two families of Boreidae and Nannochoristidae [10, 39, 40]. In the present study, we analyzed nine Mecopteran species, including *Boreus elegans* in the family Boreidae and *Nannochorista philpotti* of the family Nannochoristidae. *N. philpotti* and seven other Mecopteran species clustered together to form a clade that also includes Diptera, Megaloptera and Neuroptera, whereas *B. elegans* was in a separate clade even though it is closely related to a clade containing all members of the orders Diptera, Mecoptera, Megaloptera and Neuroptera with strongly support in all analyses (Bpp = 1.0; UFBoot = 99) (Figs. 2, 3). These results and those of several previous studies [5, 11–13] have provided insights into the phylogenetic position of the order Siphonaptera within holometabolan insects. However, they also contradict results from a few other studies [10–12]. One shortcoming of the current study is that not all lineages of fleas were included in the analyses. Therefore, further study involving more mt genomes of fleas representing all Siphonapteran families is needed to reassess the phylogeny of these families within holometabolous insects.

## Conclusions

The complete mt genome of *P. irritans* and complete coding sequences of the *C. canis* mt genome were obtained and annotated, the mt genomes of *P. irritans* and *C. canis* were compared and a phylogenetic analysis of the mt datasets was performed. This analysis revealed a clear genetic distinctiveness, demonstrating that *P. irritans* and *C. canis* are distinct species, and provided a robust phylogenetic tree that fleas are an order-level monophyletic classification. These mt genomes provide novel molecular markers for studying the taxonomy and phylogeny of fleas in the future.

## Supplementary Information

**Additional file 1: Figure S1.** PCR amplicons of the mitochondrial genome of human flea *Pulex irritans*. Amplicons are generated using the *P. irritans* primers that are included in Table S1. Abbreviations: M, DL8000 DNA marker; 1, validation_01; 2, validation_02; 3, validation_03.

**Additional file 2: Figure S2. **PCR amplicons of the mitochondrial genome of dog flea *Ctenocephalides canis*. Amplicons are generated using the *C. canis* primers showed in Table S2. Abbreviations: M, DL5000 DNA marker; 1, validation_01; 2, validation_02; 3, validation_03; 4, validation_04; 5, validation_05; 6, validation_06; 7, validation_07.

**Additional file 3: Figure S3.** 22 tRNA secondary structures from *Pulex irritans*.

**Additional file 4: Figure S4. **22 tRNA secondary structures from* Ctenocephalides canis*.

**Additional file 5: Table S1** PCR primers used to verify the mitochondrial genome of human flea *Pulex irritans*. **Table S2** PCR primers used to amplify dog flea *Ctenocephalides canis* mitochondrial genome. **Table S3** Mitochondrial genome sequences of Endopterygota insects used for phylogenetic analysis in the present study.

## Data Availability

The mitochondrial genome sequences of *Pulex irritans* and *Ctenocephalides canis* have been deposited in the GenBank database under the accession numbers ON100828 and ON109770, respectively.

## Abbreviations

AIC: Akaike information criterion
*atp*6: ATP synthase F0 subunit 6
*atp*8: ATP synthase F0 subunit 8
Bpp: Bayesian posterior probabilities
*cox*1: Cytochrome* c* oxidase subunit 1
*cox*2: Cytochrome* c* oxidase subunit 2
*cox*3: Cytochrome* c* oxidase subunit 3
*cyt*b: Cytochrome b
mt: Mitochondrial
*nad*1: NADH dehydrogenase subunit 1
*nad*2: NADH dehydrogenase subunit 2
*nad*3: NADH dehydrogenase subunit 3
*nad*4: NADH dehydrogenase subunit 4
*nad*4L: NADH dehydrogenase subunit 4L
*nad*5: NADH dehydrogenase subunit 5
*nad*6: NADH dehydrogenase subunit 6
rRNA: Ribosomal RNA
*rrn*L: Large subunit of rRNA
*rrn*S: Small subunit of rRNA
tRNA: Transfer RNA
UFBoot: Ultrafast bootstrap
