# Supplementary figures and images for: Mitochondrial phylogenomics provides insights into the taxonomy and phylogeny of fleas
Source: Parasit Vectors. 2022 Jun 22;15:223. doi: 10.1186/s13071-022-05334-3 (PMC9215091; doi:10.1186/s13071-022-05334-3)

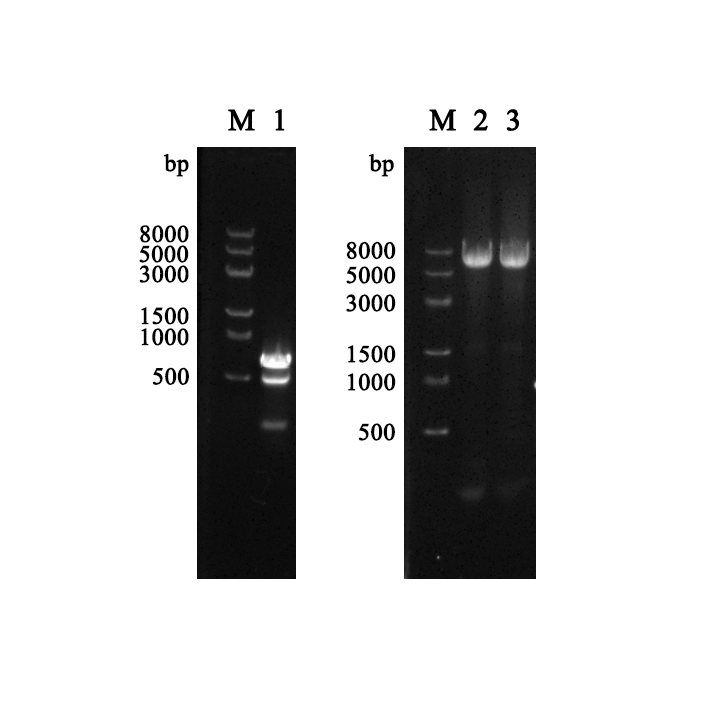

Supplement: Supplementary file 1 — Additional file 1: Figure S1. PCR amplicons of the mitochondrial genome of human flea Pulex irritans. Amplicons are generated using the P. irritans primers that are included in Table S1. Abbreviations: M, DL8000 DNA marker; 1, validation_01; 2, validation_02; 3, validation_03. [file 13071_2022_5334_MOESM1_ESM.tif]

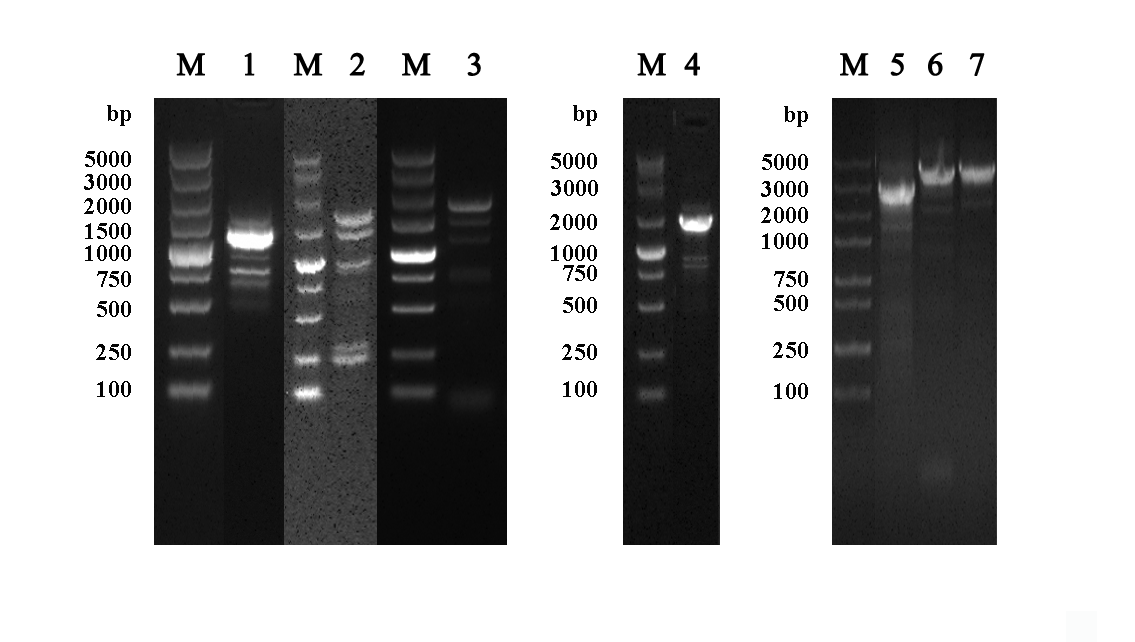

Supplement: Supplementary file 2 — Additional file 2: Figure S2. PCR amplicons of the mitochondrial genome of dog flea Ctenocephalides canis. Amplicons are generated using the C. canis primers showed in Table S2. Abbreviations: M, DL5000 DNA marker; 1, validation_01; 2, validation_02; 3, validation_03; 4, validation_04; 5, validation_05; 6, validation_06; 7, validation_07. [file 13071_2022_5334_MOESM2_ESM.tif]

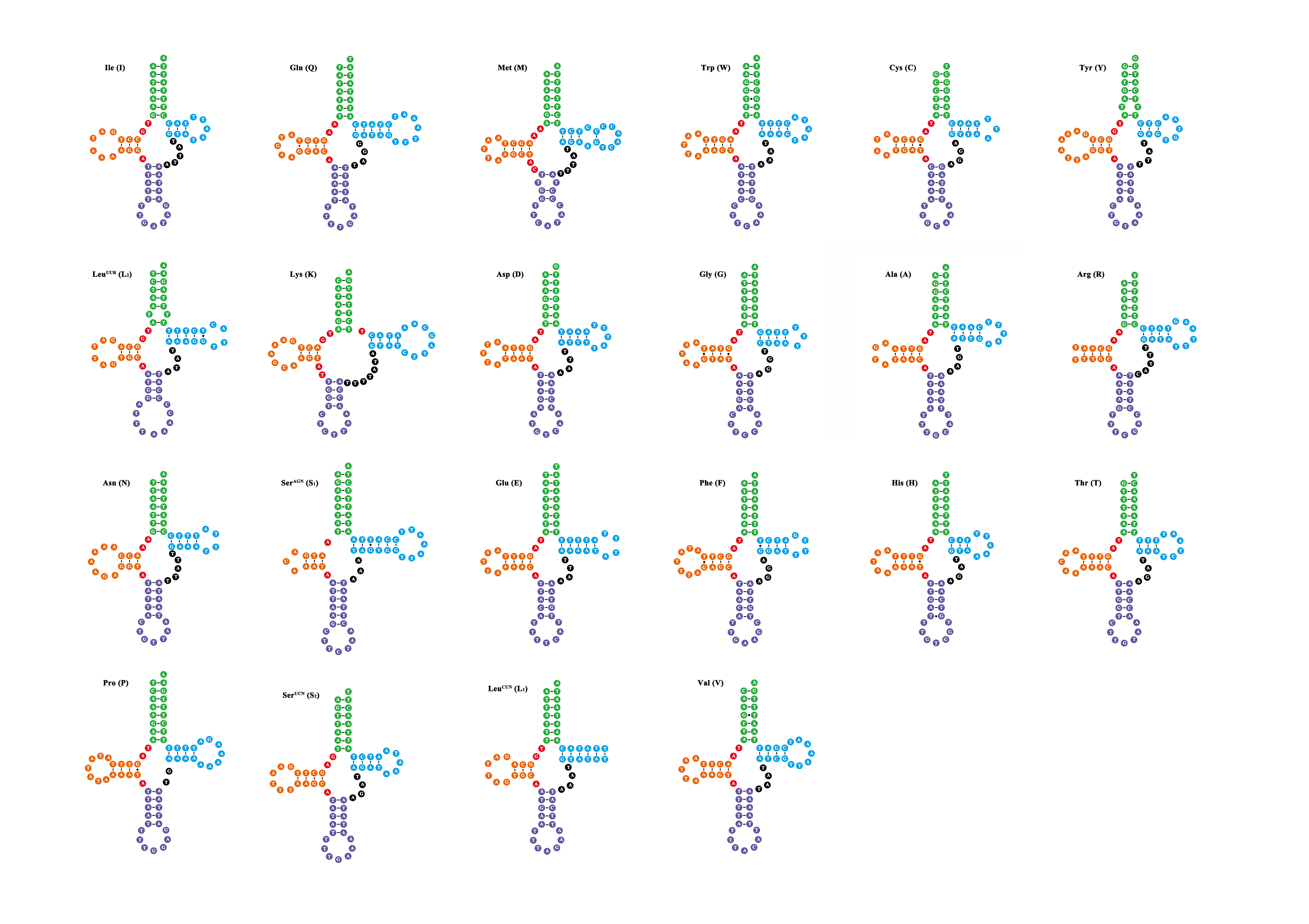

Supplement: Supplementary file 3 — Additional file 3: Figure S3. 22 tRNA secondary structures from Pulex irritans. [file 13071_2022_5334_MOESM3_ESM.tif]

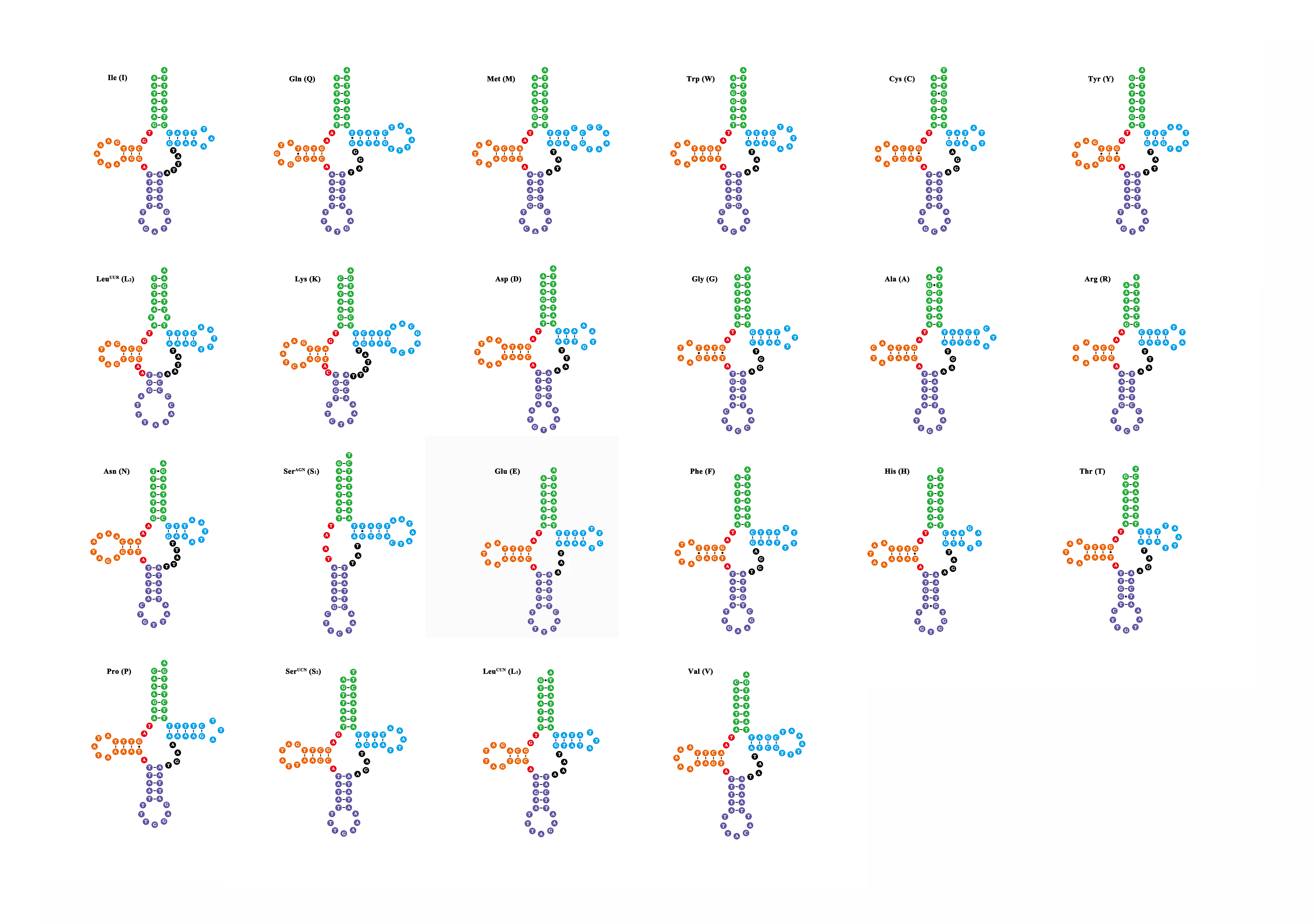

Supplement: Supplementary file 4 — Additional file 4: Figure S4. 22 tRNA secondary structures from Ctenocephalides canis. [file 13071_2022_5334_MOESM4_ESM.tif]
