# Supplementary material for: Mitochondrial phylogenomics provides insights into the taxonomy and phylogeny of fleas
Source: Parasit Vectors. 2022 Jun 22;15:223. doi: 10.1186/s13071-022-05334-3 (PMC9215091; doi:10.1186/s13071-022-05334-3)
Supplement: Supplementary file 5 — Additional file 5: Table S1 PCR primers used to verify the mitochondrial genome of human flea Pulex irritans. Table S2 PCR primers used to amplify dog flea Ctenocephalides canis mitochondrial genome. Table S3 Mitochondrial genome sequences of Endopterygota insects used for phylogenetic analysis in the present study. [file 13071_2022_5334_MOESM5_ESM.docx]

**Table S1**

PCR primers used to verify the mitochondrial genome of human flea *Pulex irritans*.

| Primer no. | Sequence (5’ to 3’) | Gene/Region |
| --- | --- | --- |
| Validation_01 | GTGCCTGATAAAAGGATTATTTTGATA | Partial I-Q-M-partial *nad*2 |
|  | TAGACAATAAGTTAAACATACTAAAGG |  |
| Validation_02 | ATCCTTTAGTATGTTTAACTTATTGTC | Partial *nad*2-W-C-Y-*cox*1-L2-*cox*2-K-D-*atp*8-*atp*6-*cox*3-G-*nad*3-A-R-N-S1-E-F-partial *nad*5 |
|  | TATCTTCTTATTTTGTTAAAAGTTCTA |  |
| Validation_03 | ACAATAATGTAACGTCACCAACCCGAT | Partial *nad*5-H-*nad*4-*nad*4L-T-P-*nad*6-*cyt*b-S2-*nad*1-L1-*rrn*L-V-partial *rrn*S |
|  | ATAGTTTAGTAGAAATGGGTTACAATA |  |

**Table S2**

PCR primers used to amplify dog flea *Ctenocephalides canis* mitochondrial genome.

| Primer no. | Sequence (5’ to 3’) | Gene/Region |
| --- | --- | --- |
| Validation_01 | ATATTTATATGTATATAACATTAATAA | Partial AT-I-Q-M-partial *nad*2 |
|  | GAGGCAAAAACTTGGATTAGAAAGTAT |  |
| Validation_02 | AAGTGCCTGAAAAAAAGGATTATTTTG | Partial I-Q-M-*nad*2-W-C-Y- partial *cox*1 |
|  | TTAATGAGGGAGGTAATAGTCAGAATC |  |
| Validation_03 | ATATAAGATTCTGACTATTACCTCCCT | Partial *cox*1-L2-partial *cox*2 |
|  | AATAGTTCAAGAATGAAGAACATCTGT |  |
| Validation_04 | TAATAATATCTCTTTTGATTCCTATAT | Partial *cox*2-K-D-*atp*8-*atp*6-partial *cox*3 |
|  | ATCATACTACATCAACAAAGTGTCAAT |  |
| Validation_05 | ATGTCACAAAATCACCCCTTTCATCTT | Partial *cox*3*-*G-*nad*3-A-R-N-S1-E-F-partial *nad*5 |
|  | CCTGTTTCAGCGTTAGTTCATTCTTCG |  |
| Validation_06 | CTAAAAAAGGAAATCCACATAATGCTA | Partial *nad*5-H-*nad*4-*nad*4L-T-P-*nad*6-partial *cyt*b |
|  | ATAGGAGTTACTAAAGGATTTGCGGGA |  |
| Validation_07 | TTGTTCAATGATTATGAGGAGGGTTCG | Partial *cyt*b-S2-*nad*1-L1-*rrn*L-V-*rrn*S-partial AT |
|  | TATATTATAAAGGGTAAAATCGTTATT |  |

**Table S3**

Mitochondrial genome sequences of Endopterygota insects used for phylogenetic analysis in the present study.

| Order | Family | Species | Size (bp) | GenBank accession number |
| --- | --- | --- | --- | --- |
| Siphonaptera | Pulicidae | *Ctenocephalides felis felis* | 20,911 | MW420044 |
|  |  | *Ctenocephalides canis* | 15,609 | MW234554 |
|  |  | *Pulex irritans* | 20,337 | MW745784 |
|  |  | *Xenopsylla cheopis* | 18,902 | MW310242 |
|  | Hystrichopsyllidae | *Hystrichopsylla weida qinlingensis* | 17,173 | NC042380 |
|  | Vermipsyllidae | *Dorcadia ioffi* | 16,785 | NC036066 |
|  | Ceratophyllidae | *Ceratophyllus wui* | 18,081 | NC040301 |
|  |  | *Jellisonia amadoi* | 17,031 | NC022710 |
| Mecoptera | Panorpidae | *Panorpa debilis* | 17,018 | NC044742 |
|  |  | *Neopanorpa pulchra* | 15,531 | FJ169955 |
|  |  | *Cerapanorpa obtusa* | 16,318 | KX091860 |
|  |  | *Neopanorpa chelata* | 16,342 | KX091857 |
|  | Boreidae | *Boreus elegans* | 16,803 | HQ696579 |
|  | Bittacidae | *Bittacus pilicornis* | 15,842 | HQ696578 |
|  |  | *Bittacus strigosus* | 15,825 | NC044741 |
|  |  | *Bittacus planus* | 15,031 | KX091849 |
|  | Nannochoristidae | *Nannochorista philpotti* | 19,092 | HQ696580 |
| Diptera | Dolichopodidae | *Xanthochlorus tibetensis* | 15,580 | NC053652 |
|  | Drosophilidae | *Drosophila melanogaster* | 19,517 | DMU37541 |
|  | Culicidae | *Anopheles gambiae* | 15,363 | NC002084 |
|  |  | *Aedes aegypti* | 16,790 | MF194022 |
|  | Psychodidae | *Phlebotomus papatasi* | 15,557 | NC028042 |
|  | Tephritoidea | *Tetanops sintenisi* | 15,763 | MT795181 |
|  | Tephritidae | *Rhagoletis pomonella* | 14,930 | MN443938 |
|  | Stratiomyidae | *Nasimyia megacephala* | 16,069 | NC054304 |
|  |  | *Tinda javana* | 15,495 | NC054251 |
|  | Sarcophagidae | *Sarcophaga crassipalpis* | 15,420 | NC026667 |
|  | Oestridae | *Gasterophilus pecorum* | 15,750 | NC029812 |
| Coleoptera | Meloidae | *Epicauta curvispina* | 15,813 | MW476523 |
|  | Cupedidae | *Tenomerga trabecula* | 16,741 | MW820160 |
|  | Elateridae | *Pyrophorus divergens* | 16,120 | EF398270 |
| Trichoptera | Stenopsychidae | *Stenopsyche tienmushanensis* | 16,049 | NC058007 |
|  | Hydropsychidae | *Hydropsyche fryeri* | 15,676 | MW413803 |
|  | Limnephilidae | *Hydatophylax nigrovittatus* | 15,048 | NC043770 |
| Lepidoptera | Oecophoridae | *Casmara patrona* | 15,393 | NC053695 |
|  | Plutellidae | *Plutella australiana* | 15,962 | NC039687 |
|  | Hesperiidae | *Malaza empyreus* | 16,103 | NC048454 |
| Hymenoptera | Formicidae | *Camponotus atrox* | 16,540 | KT159775 |
|  | Ichneumonidae | *Diadegma semiclausum* | 18,728 | EU871947 |
|  | Vanhorniidae | *Vanhornia eucnemidarum* | 16,574 | DQ302100 |
| Megaloptera | Corydalidae | *Corydalus cornutus* | 15,687 | FJ171323 |
|  |  | *Neoneuromus maclachlani* | 15,770 | MW965201 |
|  | Sialidae | *Sialis hamata* | 15,608 | FJ859905 |
| Neuroptera | Ithonidae | *Polystoechotes punctatus* | 16,036 | FJ171325 |
|  | Ascalaphidae | *Ascaloptynx appendiculatus* | 15,877 | FJ171324 |
|  | Myrmeleontidae | *Gatzara jezoensis* | 15,933 | KY364372 |
| Raphidioptera | Raphidiidae | *Mongoloraphidia harmandi* | 16,006 | FJ859902 |
|  |  | *Xanthostigma gobicola* | 13,065 | KT425093 |
|  | Inocelliidae | *Inocellia fujiana* | 14,289 | KT425085 |
| Strepsiptera | Halictophagidae | *Dipterophagus daci* | 16,255 | MW233588 |
|  | Mengenillidae | *Mengenilla moldrzyki* | 15,363 | NC018545 |
|  | Xenidae | *Xenos vesparum* | 14,519 | DQ364229 |
